# Does illicit amphetamine seizures quantity associated with amphetamine use disorder related admissions in Saudi Arabia?

**DOI:** 10.1186/s12888-023-04523-3

**Published:** 2023-01-10

**Authors:** Majed Ramadan, Enas Ghulam, Noara Alhusseini

**Affiliations:** 1 Population Health Department, King Abdullah International Medical Research Center, King Saud bin Abdulaziz University for Health Sciences, C9F6+JRH, King Abdul Aziz Medical City, Jeddah, 22384 Saudi Arabia; 2 Basic Science Department, College of Science and Health Professions, King Saud bin Abdulaziz University for Health Sciences, Jeddah, Saudi Arabia; 3 King Abdullah International Medical Research Center, Jeddah, Saudi Arabia; 4 Department of Biostatistics and Epidemiology, College of Medicine, Alfaisal University, Riyadh, Saudi Arabia

**Keywords:** Amphetamine-related disorders, Health policy, Negative binomial distribution

## Abstract

**Background:**

Illicit amphetamine-type stimulants (ATS) trafficking activities have increased substantially in Saudi Arabia over the last 10 years. In the period 2013–2017 Saudi Arabia seized the largest quantities of amphetamine at the global level. The current study examines whether the increased quantity of ATS seizures has an impact on amphetamine use disorder admissions.

**Method:**

This is an ecological study combining two datasets, the first dataset was obtained from United Nations Office on Drugs and Crime (UNODC), and the Al-Amal Hospital Electronic Health Record System in the city of Dammam, Eastern region of Saudi Arabia from 2005 to 2018. The annual incidence of patients diagnosed with amphetamine use was the dependent variable. The independent variable was the annual reported count of seized quantities of ATS in Saudi Arabia. We used a random intercept Negative Binomial model to predict the yearly count of amphetamine use disorder admission rates.

**Results:**

A total of 910 amphetamine disorder admission patients in Al-Amal rehabilitation and addiction center, and the quantity equivalent to 200 tons of ATS was seized from 2005 to 2018. The amphetamine disorder admission rate has increased from 1.33% in 2005 to 18.27% in 2018. For each one-unit increase in the amphetamine confiscated quantities, the amphetamine use disorder admission rate increased by 49 to 88%.

**Conclusion:**

The current study found that reported amphetamine seized quantities were significantly and positively associated with the increase of amphetamine use disorder-related admission rates. In 2018, both ATS seized quantities and admission rates significantly increased, nearly doubling from the previous year. Rigorous, and multidisciplinary interventional studies to evaluate factors associated with increasing abuse of ATS should be a priority for policymakers and researchers in Saudi.

## Introduction

Illicit amphetamine-type stimulants (ATS) trafficking activities have increased substantially in Saudi Arabia over the decade [1, 2]. During the previous two decades, the Middle East has been the concentration of amphetamine trafficking activities in the world. According to the United Nations Office on Drugs and Crime (UNODC) report in 2019, Saudi Arabia has seized the largest quantities of ATS at the global level [1]. In the period 2013–2017, the quantities seized by the Saudi Arabian authorities accounted for a quarter of the quantity seized worldwide followed by the United States [1]. Additionally, more than half of the quantities seized were in the East and Northeast regions of the Kingdom [1]. In response to the increasing trafficking activities and to control drugs and reduce the supply of ATS, the Saudi government like many other countries has allocated vast expenditures on criminalization and border control directed at producers, traffickers, and consumers of illegal drugs [1].

Saudi Arabia imposes extreme penalties for the import, manufacture, possession, and use of illegal drugs [2]. The purpose of spending a large proportion of their illicit drug budget on controlling the borders and market supply was to stop the following of illicit drugs in the Kingdom. Drug governmental enforcement agency in Saudi frequently and publicly shows large drug seizures as evidence of their achievement in minimizing the quantities and distribution of illicit drugs in the Kingdom. However, without examining the actual dimensional outcomes of these approaches taken by a government, we cannot confirm the effectiveness of these measures.

The large trafficking activities along with the existing demand [3] of the ATS might impact illicit ATS drug consumption behaviors and rise the harm associated with it in the Kingdom. The elevation in drug seizures is likely to reflect larger availability and scale of importation rather than a volume of change in enforcement activity [2]. A national view of the actual prevalence of Saudi drug users can be challenging due to the lack of a national drug surveillance system, and the absence of representative studies on substance use [4]. Yet several regional studies in the Southern and Central regions examined the ATS prevalence in blood among job applicants and induvial with certain health conditions. The study concluded that ATS is the most commonly abused substance, particularly among youths [2, 5]. The estimated prevalence of ATS among young people aged 17–25 was between 11 to 32% in both regions [2, 5]. These studies provide a partial reflection of how common abuse of ATS in the youth population in the Kingdom. Nevertheless, the studies do not represent the general Saudi population.

Illicit amphetamine-type stimulants (ATS) use and its associated health, and social burden continue to be a major public health challenge in Saudi Arabia. There are various types of mental, and social serious harms associated with the abuse of ATS that have a significant impact on the general population [6, 7]. A recent study in the Western region of Saudi Arabia analyzed postmortem cases and found that ATS-related mortality has increased by more than 500% in the last 3 years [7]. Additionally, nearly half of the studied cases involved violence, and approximately one-fourth of the analyzed population committed suicides [7]. This explains the substantial negative consequences of AST abuse on individuals, families, and healthcare systems as well. As substance use disorder patients have a longer length of hospital stay and more unplanned readmissions compared to the general population of hospitalized patients [8]. Which inevitably adds more strain on the healthcare system and might delay other patients seeking to find a substance use disorder treatment.

There are limited studies about AST abuse and its related policy outcomes in Saudi Arabia. Therefore, it is imperative to start with understanding how the changes in ATS seizure proportion which was the result of an existing illicit drug policy, might contribute to increased amphetamine use disorder-related hospitalizations. To our knowledge, this is the first study that examines whether the increased seizure ATS quantity has an impact on amphetamine use disorder admissions to the Al-Amal rehabilitation center. We hypothesize that an increase in illicit amphetamine seizure quantities is associated with higher amphetamine use disorder admission rate.

## Methods

### Study design, and setting

This is an ecological study combining two datasets. To estimate the annual reported seizure quantity of ATS and the annual incidence of patients diagnosed with amphetamine use disorder, it was necessary to integrate information from multiple datasets. The first dataset was retrieved from the Al-Amal Hospital in the city of Dammam, Eastern region of Saudi Arabia, including all patients who received services as an inpatient from the SUD treatment programs from the period 1/1/2005 to 31/12/2018. Al-Amal Hospital is one of the major addiction and rehabilitation centers in the region that operates under the Ministry of Health and provides free treatment and rehabilitation services.

The hospital adopted the 12 Steps Program to assist its patients in addiction recovery [9]. The 12 Steps Program started after the establishment of the center until now. The population of Al-Amal Hospital includes individuals who reside in Saudi Arabia from all regions. Individuals aged 18 or older who were diagnosed with amphetamine use disorders and hospitalized between the years 2005 to 2018 were included in the analysis. This study received approval and a waiver of informed consent from the Ministry of Health IRB. Informed consent was waived because data were deidentified.

The second dataset was obtained from United Nations Office on Drugs and Crime (UNODC) using the Saudi annual reported ATS seizure quantities from the year 2005 to 2018. UNODC collects the data annually from all United Nations members to enhance the comparability of drug and crime statistics at the international level and to support countries in their efforts to produce national statistics on drug use and crime rates [10]. The data is a public use file available for researchers at their website [10].

### Study variables and data source

Al-Amal Hospital dataset was the aggregated data obtained from the health record system of Al-Amal Hospital using the International Classification of Diseases (ICD-9-CM 305.70 and ICD-10-CM codes F15.10) for each admission and then confirmed with written clinical primary and secondary diagnoses. We included patients diagnosed with amphetamine use disorder during their admission from 2005 to 2018 for ages 18 or older. Then, combined the two-dataset using the year as a common matching variable. The annual incidence of patients diagnosed with amphetamine use disorders (DSM-IV abuse or dependence) served as a dependent variable. For our independent variable, we used the annual reported count of ATS seizure quantities per kilogram in Saudi Arabia.

### Statistical analysis

For initial descriptive statistics, we constructed rates for the frequency of amphetamine hospitalization as a percentage of all amphetamine disorder admissions from 2005 to 2018 as the dominator. We also constructed rates for amphetamine seized quantities as a percentage of the total seized count for the 14 years from 2005 to 2018. In our model, we combined two datasets from UNODC and Al-Amal Hospital. The resulting dataset had an observation for each amphetamine use disorder hospitalization in the year and the annually reported seizure quantities of ATS.

We used a random intercept Negative Binomial model to predict the yearly count of amphetamine use disorder admission accounting for the overdispersion of count data. We further used robust standard errors to correct the effect of overdispersion and heteroscedasticity on the standard errors [11].

$${\text{ASQC}}_{\text{j}\text{i}}=\exp\ \left({\beta}_{0\text{i}}+{\beta}_1\;{\text{ADAC}}_{\text{j}}\;\text{Time}\;\textrm{i}+{\beta}_2\;{\text{Age}}_{\text{j}}+{\beta}_3\;\text{Time}\ {\text{i}}_{\text{j}}+\varepsilon \text{ij}\right)$$

Amphetamine seizures quantity count (ASQC) a random intercept (i1,..,14) in the year (j1 …,14) are modeled as a function of the coefficient β1–3 representing amphetamine disorder admission count (ADAC), and age in year j. We included the interaction term between ADAC and time to estimate the incidence rate ratio of ASQC relative to ADAC for each year. All statistical tests were 2-sided, and findings were considered statistically significant at *P* < .05. All analyses were conducted using SAS statistical software version 9.4 (SAS Institute Inc. Cary, NC).

## Results

The trend in amphetamine disorder admission rates is shown in (Table 1). The amphetamine disorder admission rates have increased from 1.33% in 2005 to 18.27% in 2018. Similarly, the ATS seizure quantity increased from 4.11% in 2005 to 14.12% in 2018. (Table 1) This study examined a total of 910 amphetamine disorder admission patients in the Al-Amal rehabilitation and addiction center and an ATS seizure quantity of 178,572.02 which is approximately equivalent to 200 tons from 2005 to 2018. (Table 1).

**Table 1:** Trend in amphetamine reported quantities rate amphetamine use disorder admission rate

	Amphetamine use disorder admission rate ratio^**2**^ N(%)	Amphetamine reported sized quantities rate ^**1**^ N(%)
	Incident Rate	Incident Rate
**Total**	903 (100)	178,572.02 (100)
**2005**	12 (1.33)	7342.42 (4.11)
**2006**	41 (4.54)	9459.21 (5.29)
**2007**	42 (4.65)	12,832.37 (6.88)
**2008**	51 (5.64)	10,403.11 (7.18)
**2009**	50 (8.19)	12,551.5 (7.02)
**2010**	49 (5.53)	8799.749 (4.92)
**2011**	51 (5.64)	11,452.31 (6.41)
**2012**	67 (7.41)	8260.17 (4.62)
**2013**	74 (8.19)	9705.52 (5.43)
**2014**	75 (4.65)	17,112.68 (9.58)
**2015**	68 (8.31)	11,363.23 (6.36)
**2016**	64 (8.31)	17,820.95 (9.98)
**2017**	94 (7.09)	16,275.49 (9.11)
**2018**	165 (18.27)	25,192.81 (14.12)

^1^quantity per kilogram

**Fig. 1:**
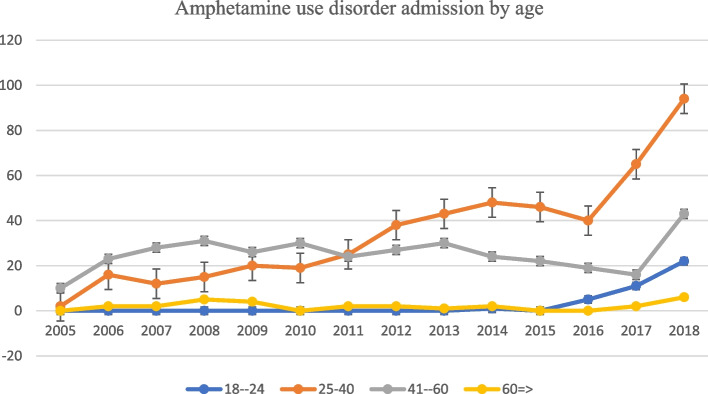
Amphetamine use disorder admission by age

The age group 25–40 years old showed a consistent increase in amphetamine disorder admission rates across the study period. (Fig. 1) In 2018 alone, more than half of the amphetamine use disorder admitted patients (55.29%) were in the age group 25–40 years old. From 2013 to 2016 there was a slight decrease in the amphetamine use disorder admission rate for the age group 41–59 years old from 8.49% in 2013 to 5.38% in 2016. (Fig. 1).

**Fig. 2:**
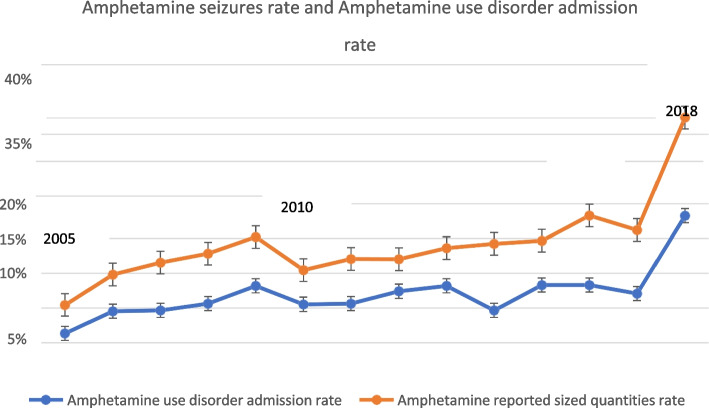
Amphetamine seizures rate and Amphetamine use disorder admission rate

The regression slope of amphetamine uses disorder admission rate increased over time significantly. (Fig. 2) Similarly, there was a statistically significant increase in ATS seizure quantity across the years 2005 to 2018. (Fig. 2).

In the age-adjusted negative binominal model results are presented as incident rate ratios and showed a statistically significant association between amphetamine use disorder admission rate and ATS seizures quantity for the years 2009, 2010, 2012, 2013, 2015, and 2018 (IRR 1.16; 95% CI = 0.02–1.99; *P* = .03, IRR 1.12; 95% CI = 0.021–2.51; *P* = .04, IRR 1.51; 95% CI = 0.073–2.62; *P* = .03, IRR 1.6; 95% CI = 0.042–2.62; *P* = .01, IRR 1.31; 95% CI = 0.31–2.47; *P* = .01, IRR 1.229; 95% CI = 0.078–0.26; *P* = .002), respectively. (Table 2) The ATS seizures quantity was a significant predictor for amphetamine use disorder admission rate. For each one-unit increase in ATS seizures quantity, the amphetamine use disorder admission rate increased by 49 to 88%. (Table 2).

**Table 2:** Negative Binomial Regression Model for association between amphetamine reported quantities rate amphetamine use disorder admission rate

	Amphetamine use disorder admission incident rate ratio			
	Estimate IRR^**2**^	Standard Error	95% Confidence Intervals	***P*** value^**1**^
**Annual amphetamine reported sized quantities**				
**2005**	0.32	0.84	(−2.65, 0.65)	0.23
**2006**	0.85	0.87	**(**0.09, 2.51)	0.03
**2007**	0.67	0.61	(−0.41, 1.65)	0.23
**2008**	0.78	0.52	(− 0.242, 2.1)	0.12
**2009**	1.16	0.59	(0.02, 1.99)	0.03
**2010**	1.12	0.51	(0.021, 2.51)	0.04
**2011**	0.9	0.63	(0.022, 2.12)	0.05
**2012**	1.51	0.54	(0.073, 2.62)	0.03
**2013**	1.6	0.65	(0.042, 2.62)	0.05
**2014**	0.48	0.61	(−0.71, 0.94)	0.78
**2015**	1.31	0.42	(0.31, 2.47)	0.01
**2016**	0.83	0.55	(−0.35, 1.11)	0.31
**2017**	0.77	0.37	(−0.32, 1.31)	0.12
**2018**	1.29	0.41	(0.078, 0.26)	0.002

^1^Negative Binomial regression model

^2^Incident rate ratio

## Discussion

Amphetamine use disorder-related hospitalizations are rising in the Eastern region of Saudi Arabia with rates increasing nearly 18 times from 2005 to 2018. In consistency with previous studies, our study revealed that the 25–40 age group amphetamine-related admission rate has largely and consistently increased from 2005 to 2018 [12]. The current study found that reported ATS seizure quantity was significantly and positively associated with the increase in amphetamine use disorder admission rates, which rejects our null hypothesis. This association is more apparent toward the end of the study period, particularly from 2013 to 2017 when the largest quantity of ATS was seized by the authorities.

In 2018, both ATS seizure quantity and admission rates significantly increased, nearly doubling from the previous year. However, there was a statistically insignificant fluctuation in a few years of the study period, particularly between the years 2005 to 2008. This might be attributed to geopolitical reasons, as the civil war in Syria started in 2011 leading to a spike in illicit ATS manufacturing [1]. According to the UNODC report, in the last 10 years, large quantities of illicit amphetamine-type stimulants seized in Saudi Arabia were manufactured in the Syria Arab Republic, and Lebanon [1].

One theory that might explain the link between amphetamine-related admissions and ATS seizure quantity is that the law enforcement war against drug trafficking doesn’t necessarily mean a reduction in drug supply [12, 13]. On the contrary, it could reflect the unseized quantities entered the kingdom, which means more ATS supply, higher availability, and elevation of negative consequences such as rising crime rate, violence, and hospitalizations [13, 14]. Increased amphetamine-related admissions are likely due to rising in amphetamine use and availability. As an Australian study concluded that fluctuations in amphetamine availability explained half of the variation in amphetamine-related admissions [15]. In general, increasing the severity of law enforcement toward drug trafficking associated with dealing drugs raises for all types of illicit drugs [14, 15]. In the U.S. during Nixon’s presidency, policymakers announced a war against drug trafficking to achieve their goal of a drug-free world. Years later, studies found that cocaine and heroin prices have fallen substantially during a period of the most increased movement in law enforcement against drug trafficking [16]. Another significant aspect that might interpret the relationship of amphetamine seized quantities with the increase of amphetamine use disorder-related admission rates is illicit drug potency. Overall, the purity and potency of illicit drug material have consistently increased over time [17]. In the recent World Drug Report in 2021, they revealed that organized crime groups succeeded in manufacturing highly potent amphetamine and making it available in the illicit drug market [18]. Therefore, it is likely that the seized quantities of ATS in the last decade contain more potent ATS than ever before which in return might result in a larger number of users with a higher risk of addiction who might seek treatment.

Saudi Arabia is considered a major target for many illicit drug traffickers in the world such as traffickers in Eastern Europe, and Southeast Asia [1]. In response to the rising trafficking activities, the authorities represented by the General Directorate of Narcotics Control (GDNC) in Saudi have initiated a strict prohibition against the possession, dealing, and consumption of illicit substances [3]. The purpose of this initiation was based on the theory that the vigorous enforcement of laws prohibiting drug production, distribution, and use will eventually break the cycle of the supply and demand of drugs, and subsequently exterminate the illicit drug market. It has been demonstrated in many regions in the world like North America, Latin America, and Europe that law enforcement approaches concentrate on arrests and punishment toward producers, traffickers and users have been failing in reducing the scale of illicit drugs in the markets [14, 19–21]*.* Thus, the GDNC strategy might not have been successfully achieving its goal of weakening the drug market in the Kingdom. However, no previous studies have been conducted in Saudi to confirm or reject this hypothesis.

Rigorous, and multidisciplinary interventional studies to evaluate the factors associated with the increasing abuse of ATS should be a priority for policymakers and researchers in Saudi Arabia. Policymakers in Saudi are highly encouraged to start investing heavily in research and investigation into the effect of different policies and programs implemented in the Kingdom. Meanwhile, harm reduction efforts should be applied to enhance the awareness level of amphetamine abusers and minimize amphetamine related-fatal risks. This can be accomplished through widespread educational and interventional programs on the risk associated with amphetamine use targeted toward the community, especially high-risk age groups. Additionally, encouraging and providing adequate culturally sensitive treatment services for individuals suffering from amphetamine use disorder should be considered. These collective efforts will contribute to a movement of breaking the taboos, establish an open debate, and promote policies that effectively decrease the number of ATS users, and would eventually prevent potential ATS-related harms.

There are several limitations to this study. First, due to the nature of the observational study, only correlations but no causal relationships can be determined between sized ATS and amphetamine use disorder admission rates. Second, the study is subject to ecological fallacy, conclusions are inferred about individuals from the results of aggregate data assuming all individual members of the two datasets have the average characteristics of the group as a whole.

## Conclusion

Amphetamine use disorder-related hospitalizations are rising in the Eastern region of Saudi Arabia with rates increasing nearly 18 times from 2005 to 2018. The current study found that reported ATS seizure quantities were significantly and positively associated with the increase in amphetamine use disorder-related admission rates. In 2018, both ATS seizure quantities and admission rates have substantially increased, nearly doubling from the previous year. Rigorous, and multidisciplinary interventional studies to evaluate factors associated with increasing abuse of ATS and other illicit drugs should be a priority for policymakers and researchers in Saudi.

## Data Availability

The data that support the findings of this study are not publicly available. Restrictions apply to the availability of these data, which were used under license for this study. Data are available [at Al-Amal Hospital, and Ministry of Health https://www.moh.gov.sa/en/Ministry/Forms/Studies-and-Researches/Pages/default.aspx] with the permission of [Ministry of Health].
